# Metagenomics to characterize sediment microbial biodiversity associated with fishing exposure within the Stellwagen Bank National Marine Sanctuary

**DOI:** 10.1038/s41598-022-13409-5

**Published:** 2022-06-09

**Authors:** Spencer A. Bruce, Semra A. Aytur, Cheryl P. Andam, John P. Bucci

**Affiliations:** 1 Department of Biological Sciences, University at Albany, State University of New York, Albany, NY 12222 USA; 2 Department of Health Management & Policy, University of New Hampshire, Durham, NH 03824 USA; 3 School of Marine Science & Ocean Engineering, University of New Hampshire, Durham, NH 03824 USA; 4 Marine Microverse Institute, Kittery Point, ME 03905 USA

**Keywords:** Metagenomics, Bioinformatics, Biodiversity, Marine biology

## Abstract

Microbes in marine sediments constitute a large percentage of the global marine ecosystem and function to maintain a healthy food web. In continental shelf habitats such as the Gulf of Maine (GoM), relatively little is known of the microbial community abundance, biodiversity, and natural product potential. This report is the first to provide a time-series assessment (2017–2020) of the sediment microbial structure in areas open and closed to fishing within the Stellwagen Bank National Marine Sanctuary (SBNMS). A whole metagenome sequencing (WMS) approach was used to characterize the sediment microbial community. Taxonomic abundance was calculated across seven geographic sites with 14 individual sediment samples collected during the summer and fall seasons. Bioinformatics analyses identified more than 5900 different species across multiple years. Non-metric multidimensional scaling methods and generalized linear models demonstrated that species richness was inversely associated with fishing exposure levels and varied by year. Additionally, the discovery of 12 unique biosynthetic gene clusters (BGCs) collected across sites confirmed the potential for medically relevant natural product discovery in the SBNMS. This study provides a practical assessment of how fishing exposure and temporal trends may affect microbial community structure in a coastal marine sanctuary.

## Introduction

Microbial communities on the ocean seafloor contain vast numbers of undiscovered bacterial species^1^ and harbor more biodiversity than previously recognized^2^, potentially in response to multiple stressors^3^. Compared to bacteria living in the water column, sediment bacteria at the surficial layer tend to be more diverse in deep-sea versus shallow continental regions^4^. These microbes also drive essential benthic processes such as nutrient regeneration, organic matter oxidation, removal of toxins, and production of biosynthetic compounds^5^. Continental shelf sediment ecosystems are unique since they are exposed to terrestrial-based sources of nutrients and contaminants as well as variations in regional climate conditions^6^. The Stellwagen Bank National Marine Sanctuary (SBNMS), located off the coast of Massachusetts, offers an ideal model to explore sediment microbial taxa, since it is a vital habitat for threatened marine species and serves as a working sanctuary, providing access to commercial fisheries. The sanctuary is a patchwork of benthic substrate types that are associated with a diverse group of ecosystems. The benthic habitat supports a variety of fish and invertebrate species, such as sand lance, cod, haddock, cephalopods, crustaceans, and annelids^7^.

The Gulf of Maine (GoM) system is located in the Atlantic continental region of the United States. The GoM is a vital ecosystem that warrants greater understanding. It lies within a geologically passive margin, which has allowed large areas of thick sediment to accumulate, resulting in a relatively shallow shelf. Biodiversity conservation is a key management priority for the SBNMS and for marine sanctuaries worldwide^7^, yet time-series assessments of microbial taxa are lacking^8^. There is an urgent need to characterize this vital habitat, especially in protected and fished areas, by reconstructing community profiles to better understand the effects of anthropogenic and environmental changes. Monitoring studies of food web populations in the SBNMS over the past ten years suggest that there are measurable impacts on habitat quality conditions that may threaten benthic communities, including microbial species^9^, possibly related to the impacts of bottom gear used in commercial fishing. Mobile, bottom-tending fishing gear can directly affect the surface sediment where the more recent microbial communities reside by mechanically altering the structural characteristics and biological components of the seabed, which is a concern for maintaining habitat integrity^10,11^.

Marine sediment microbial communities near continental shelf regions and similar deep-seafloor sediment ecosystems (below 200 m) are not well documented^12,13^. Previous reports evaluating the benthic community on a small spatial scale suggest that a relatively high level of biodiversity supports the food web within the SBNMS^14^, especially when compared to similar habitat in the deep sea^15^. A key distinction between these two habitats is that nearshore sediment communities can experience more frequent exposure to fishing, and the use of trawls and dredges is considered one of the primary activities that adversely influences the physical and biological characteristics of benthic habitat integrity^16^. The present report focuses on improving current knowledge of the sediment microbial community structure in the SBNMS in relation to fishing exposure and temporal trends, which may impact abundance and diversity.

According to a prior report, there are diverse species of benthic invertebrates within SBNMS, including communities of sponges and anemones considered rare within the GoM.^7^ However, sanctuary regulations allow recreational and commercial fishing in some areas, designating the SBNMS as a working sanctuary. Intermittent exposure to commercial fishing activity, including many types of gear (dredges, trawlers, fixed traps), may alter benthic habitats within the SBNMS^17^. Reports suggest that the biodiversity of the food web remains relatively healthy^18^; however, concerns have been raised about this status relative to the sediment habitat.

Advances in sequencing of microbial species has enabled the study of metagenomes from environmental samples^19^. Research has shown that microbial communities harbor biosynthetic gene clusters (BGCs) with potential utility for medicinal purposes^20,21^. Marine sanctuaries such as the SBNMS serve as a living resource, supporting productive fisheries and providing health-promoting ecosystem services through their potential to host medically relevant bacterial strains that can be used in natural products. Approaches such as 16S ribosomal RNA sequencing show that sediment type may influence microbial community composition^22^. Traditional clonal cultures and gene sequencing, such as 16S rRNA, produce a profile of diversity in an environmental sample, although these methods can exclude a vast majority of biodiversity^23^. Conversely, innovative whole metagenomic sequencing (WMS) techniques are valuable in assembling partial genomes to assess less abundant taxa^24^, which improves diversity estimates across an entire community^25^. To a considerable degree, metagenomics provides greater coverage in the estimation of the organisms in a sample, thereby providing a powerful tool to characterize unculturable marine sediment bacteria and establish a baseline for ecological monitoring studies^26^.

The objectives of this study were to characterize the taxonomic relative abundance and biodiversity of sampling sites within the SBNMS that have been exposed to different levels of fishing activity. A metagenomic database was constructed consisting of microbial taxa by geographic site and fishing exposure status from 2017 to 2020. Sites within the SBNMS designated as closed to fishing were compared to those exposed primarily to fishing with fixed gear, designated ‘minimal’ to ‘moderate’ fishing activity according to recent reports^9^. An additional aim was to assess bacterial species with the highest potential for harboring medically relevant biosynthetic gene clusters (BGCs) encoding secondary metabolites such as nonribosomal peptide synthetases (NRPS), which have antibacterial and antitumor properties.

## Methods

### Study area and sampling sites

**Figure 1:**
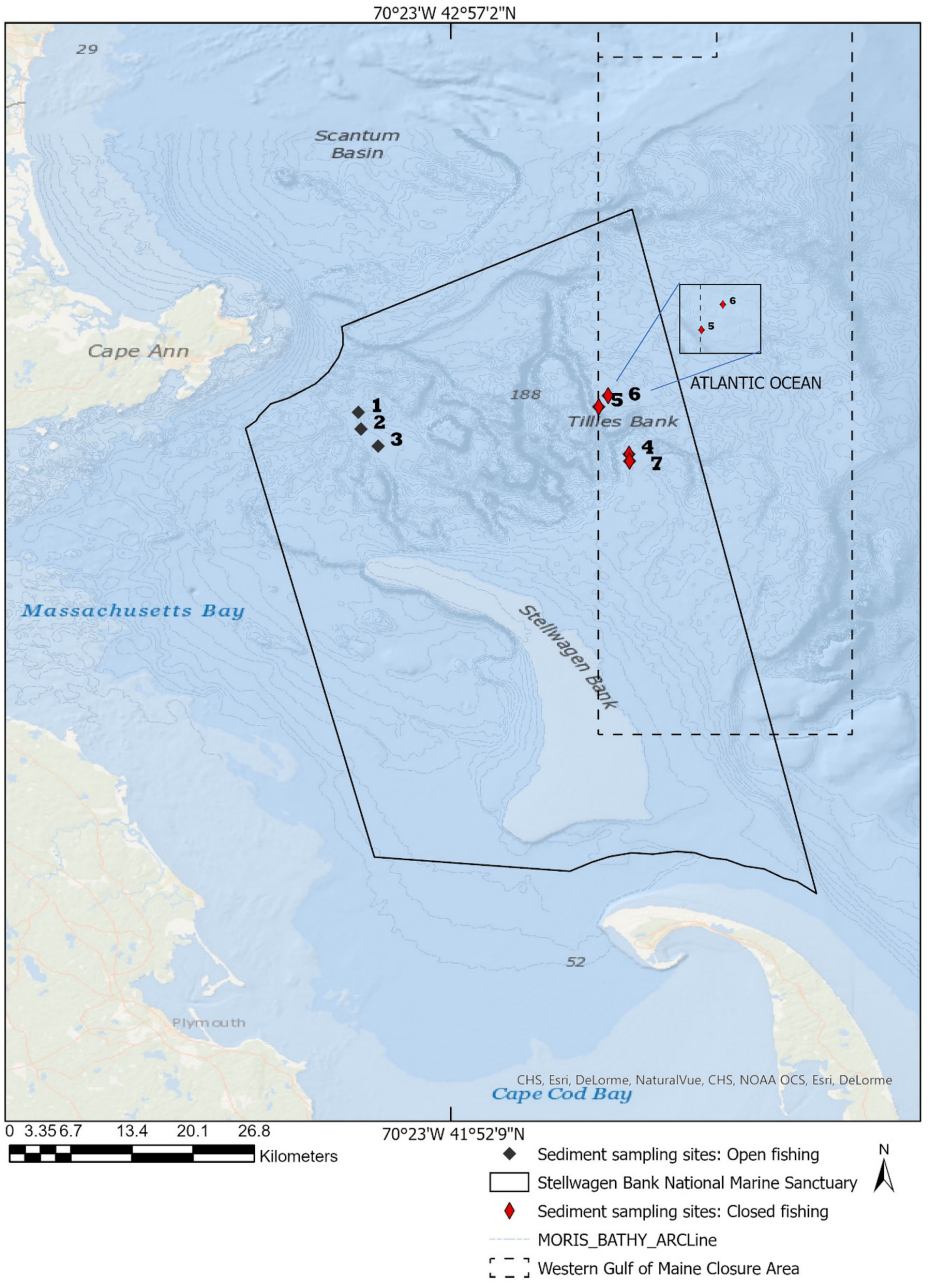
Map of sampling sites. Black symbols indicate open to fishing. Red symbols indicate closed to fishing, 30 m within the closure boundary. Note: Map was created by the authors using ArcGIS Pro 2.8 based from the NOAA Coastal Survey Data (https://www.nauticalcharts.noaa.gov/data/gis-data-and-services.html).

The SBNMS study area is under permit from the National Oceanic and Atmospheric Administration (NOAA). The sanctuary is an 842-square-mile federal marine protected area that stretches from Cape Ann to Cape Cod, Massachusetts, USA. Sampling locations included sites 1 (42.566889°, − 70.486222°), 2 (42.550362° − 70.483694°), 3 (42.533667°, − 70.466763°), 4 (42.525639°, − 70.219722°), 5 (42.572306°, − 70.249528°), 6 (42.583333°, − 70.240278°), and 7 (42.518889°, − 70.219167°) (Fig. 1). The sampling scheme was designed in collaboration with SBNMS scientists. Sites 1, 2 and 3 were designated open to recreational and commercial fishing activities. Sites 4, 5, 6, and 7 were designated as closed to fishing and are part of the Western Gulf of Maine Groundfish Closure Area, where fishing activities are prohibited^27^. The depth ranged from 80 to 120 m from the sea surface to the benthic surface below the photic zone.

### Sediment collection and processing

From 2017–2020, sediment samples were collected using a Van Veen grab from the surficial benthic layer (top 20 cm at the water column interface). The surficial layer at the sediment-water interface represents the more recent microbial community^12,28^. During the summer season, we sampled sites 1 (2017, 2019) 2 (2017), 3(2017,2018,2019,2020), 4 (2018), 5 (2018), 6 (2019), and 7 (2019). One sediment grab from each site was collected and measured for grain size by particle sieve analysis. From each grab collected across the seven geographic sites, a total of 14 sediment samples were analyzed. Approximately 30 g of sediment were transferred from the grab to a sterile 50 mL Falcon™ tube. The collection tubes were immediately placed on ice in a container onboard until returned to the laboratory. DNA was then extracted from 0.30 g of sediment samples using a PureLink® DNA kit (Thermo Fisher Scientific™). The DNA extract was pooled based on sample replicates, and concentrations were quantified using a Qubit 3.0 fluorometer (Thermo Fisher Scientific). The sample DNA was stored at − 20 °C until sequencing.

### Sequencing

Whole metagenome sequencing for taxonomic profiling was performed at the University of New Hampshire, Hubbard Center for Genome Studies (Durham, NH). This method delineates a majority of random fragments from a diverse community sediment sample in a shotgun approach. This technique results in greater sequencing coverage of a wide range of organisms. Library preparation included ligation adapters designed to target short paired ends (2 × 250 bp) and performed on an Illumina™ HiSeq® 2500 using a Cluster Kit v4 (San Diego, CA, United States). Large insert (500 bp) libraries were then created to account for the diversity in the samples and to maximize high-quality assembly of all microbial taxa.

### Metagenomic data processing, taxonomic classification, and abundance

All raw sample data underwent initial processing using the metaWRAP pipeline v1.3.2^29^, employing the metaWRAP-Read_qc module. Read quality control (QC) was first carried out using FASTQC v0.11.8 to generate pre-QC reports^30^. All reads were then subjected to trimming index removal using TrimGalore v0.5.0^31^, and a post-QC report was generated for comparison. Replicates of trimmed and decontaminated forward and reverse reads from each sample site, collected on the same date, were combined for all downstream analysis resulting in a forward and reverse FASTQ file for each. Metagenomic assemblies were then carried out using the metaWRAP-Assembly module employing SPADES v3.13.0^32^, utilizing the metaSPAdes option flag, and assembly reports were generated using the program QUAST v5.0.2^33^. The results of the Kraken2 analysis were processed with Bracken v2.6 (Bayesian re-estimation of abundance after classification with Kraken)^34^, which allows for the estimation of abundance at multiple taxonomic levels. The abundance of the top five groups at the phylum, class, and genus levels for each sample site was plotted using the ggplot2 v3.3.5 package in R^35^. The metaWRAP-Kraken2 module was then run on both the combined trimmed and decontaminated reads and the assembly produced by SPADES. Running Kraken on the reads was carried out to determine the taxonomic composition of the communities, while running Kraken on the assemblies was carried out to determine which taxonomic groups were assembled better than others. This was done for each sample site at each date to classify reads at multiple taxonomic levels utilizing the complete NCBI RefSeq bacterial genome library. Subsampling was carried out at the level of 3,500,000 reads to account for differences in the number of reads among sample sites. Additionally, combined abundance across all sample sites was calculated using the combine_bracken_outputs.py script and pooled based on the combined trimmed reads only. The output was visualized in a Sankey diagram with the web application Pavian^36^.

### Genome binning and the detection of BGCs

To identify high-quality metagenomes (MAGs) across sample sites, reads and coassemblies were binned using the metaWRAP Binning module employing 3 different commonly used algorithms: MaxBin 2.0^37^, CONCOCT^38^, and MetaBAT 2^39^. The resulting bins for each sample site were consolidated into a final bin set with metaWRAP’s Bin_refinement module, retaining only bins that were at least 50% complete exhibiting less than 10% contamination as determined by CheckM^40^. The resulting MAGs were concatenated and analyzed utilizing Kraken2 to determine the taxonomic classification across all sample sites and visualized as a kronagram using KronaTools^41^. High-quality MAGs at the level of each sample site were also functionally annotated using the program Prokka^42^. Finally, annotated genomes were screened for BGCs using the antiSMASH™ pipeline^43^, and the results were plotted using ggplot2^44^. A radar plot was also created using ggRadar to visualize the proportion of BGCs detected at each site in each year^45^. Computations were carried out using the University at Albany’s high-performance computing cluster using the default program parameters unless otherwise specified above.

### Analysis of species diversity and abundance in the context of fishing exposure

Diversity analyses were first carried out using the R package vegan v2.5–7^46^, and plots were made with ggplot2. The observed, Shannon-Weiner diversity index (*H'*), Simpson (measure of the probability that randomly selected individuals from a sample will be the same) InvSimpson and Fisher alpha diversity measures were calculated across all samples using the abundance estimates produced by Bracken. These measures were then plotted in the context of open and closed fishing status. The Shannon index measures the relative abundance of species present in a community^12^. Additional plots were created to illustrate the level of fishing exposure measured on an ordinal scale (delineated as 0 (none), 1 (low), and 2 (moderate)). Next, species richness (total number in a community) was calculated and plotted in the context of these variables. Nonmetric multidimensional scaling (NMDS) using R^47,48^ and generalized linear models (GLMs)^49^ were used to elucidate the relationships of species richness and abundance with fishing exposure over time, adjusting for sediment grain type. NMDS and GLM were chosen because these methods are robust to departures from normality and can account for nonlinear assumptions. Generalized linear models introduce a link function around the combination of the explanatory variables, so that non-normal and discrete distributions of Y can be fitted within this model class. In GLMs, the response variable y_i_ is assumed to follow an exponential family distribution with mean μ_i_, which can be a linear or nonlinear function of x_iTβ_. In our GLM, a logit link function was used with the zero-inflated negative binomial distribution to model abundance. The inverse gaussian distribution with a log link was used in models of biodiversity.

### Fishing exposure levels

For the current study, an ordinal fishing exposure level variable was derived from NOAA fisheries vessel monitoring system (VMS) data in a report^7^, which uses codes to categorize a range of gear types^50^. The VMS data provide a spatial representation of commercial fishing, which is used as a standard method to account for mobile bottom-tending gear^51^. VMS uses integrated global positioning systems installed on vessels to transmit, via satellite, the vessel’s location. The classifications were labeled according to the following scale: 0 = no fishing exposure/closed to fishing; 1 = low fishing exposure; 2 = moderate fishing exposure.

### Water quality

Water quality parameters (i.e., temperature, salinity, dissolved oxygen, and chlorophyll a) were documented from the Northeastern Regional Association of Coastal Ocean Observing Systems (NERACOOS) proximal to the open-to-fishing sampling sites^52^. These data provide contextual information relevant to the sampling area as a whole, although the location of the buoys does not permit granular site-level analysis. Based on the NERACOOS data, we examined the daily average standard deviations of 15-min temperature recordings against the times that we sampled, and verified that the value was within 1 SD of the seasonal mean for that year. This allowed us to confirm that there were no outliers or extremely hot or cool sampling days with respect to temperature variations.

## Results

### Metagenomic data summary and community composition

**Figure 2:**
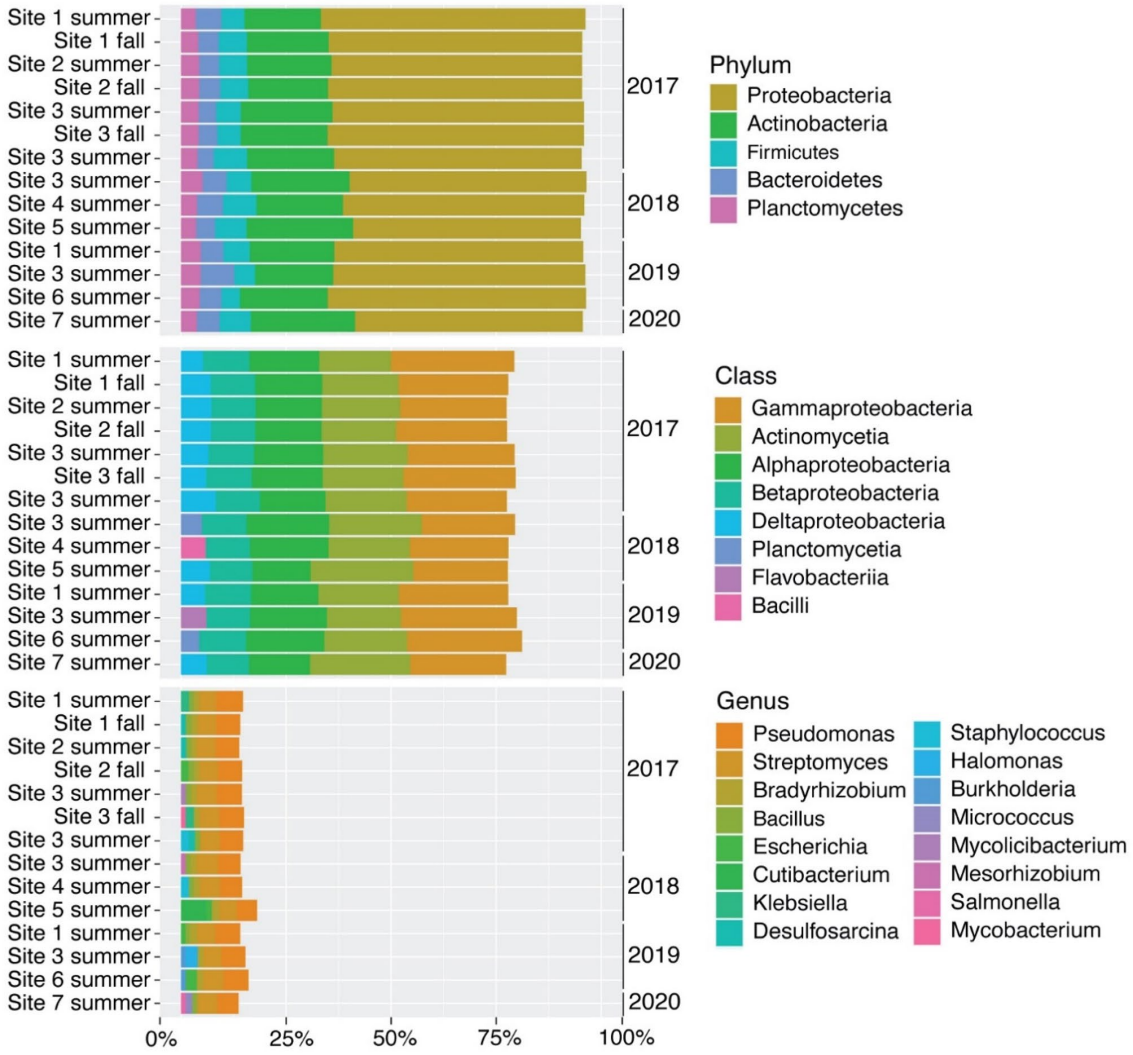
Stacked bar plots showing relative abundance for the top five groups at each sample site and at each taxonomic level: phylum, class, and genus.

**Figure 3:**
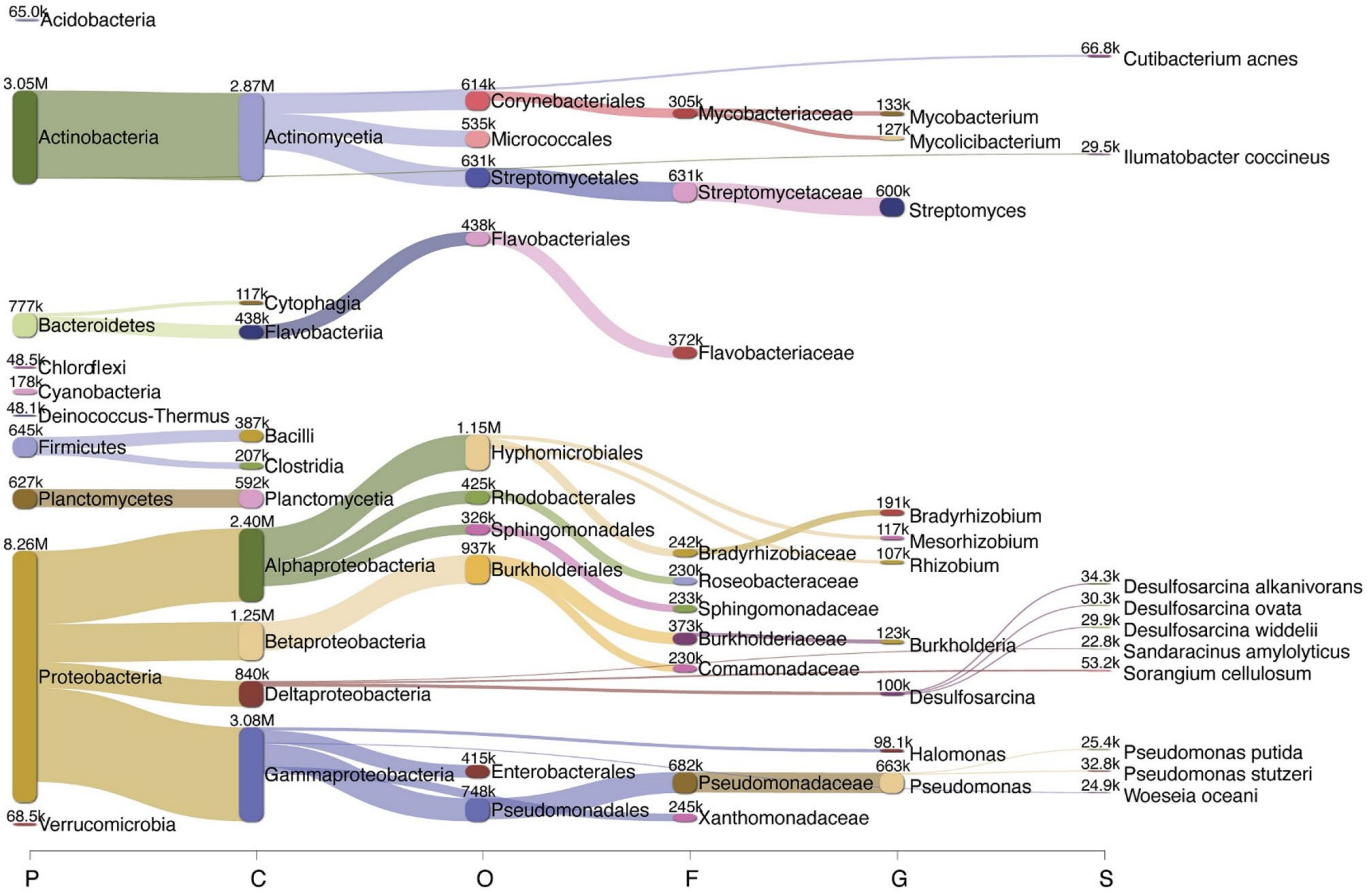
Sankey diagram produced by Pavian with the total number of corrected reads as estimated with Bracken for all sites combined at the level of phylum, class, order, family, genus and species.

Protobacteria was the dominant phylum across all sites, representing 54 to 63% of the bacteria identified (Fig. 2). Actinobacteria was also prevalent, representing 18% to 25% of the bacteria identified, whereas Firmicutes, Bacteroidetes and Planctomycetes made up a smaller proportion (ranging from 4–8%, 4–8%, and 4–5%, respectively). At the class level, Gammaproteobacteria was the most prevalent across all sites, representing from 22–30% of the bacteria identified, followed closely by Actinomycetia (17–24%) and then Alphaproteobacteria (14–20%). At the genus level, abundance estimates exhibited far more diversity, although all sites were dominated by a relatively small proportion of *Pseudomonas* (5–6%) and *Streptomyces* (4–5%) (Fig. 2). Abundance estimates produced by Bracken combinations were visualized across phyla, classes, orders, families, genera and species (Fig. 3). The abundance estimates detected 5986 species across the 14 sample sites with more than 10 reads, ranging from 5123–5441 species depending on the site examined. The mean number of species with more than 10 reads identified was 5268. Analyses showed that sites 7 and 4 exhibited the highest abundance of species, while site 5 exhibited the lowest (Table S1).

### Comparisons of species diversity by site and fishing exposure

**Figure 4:**
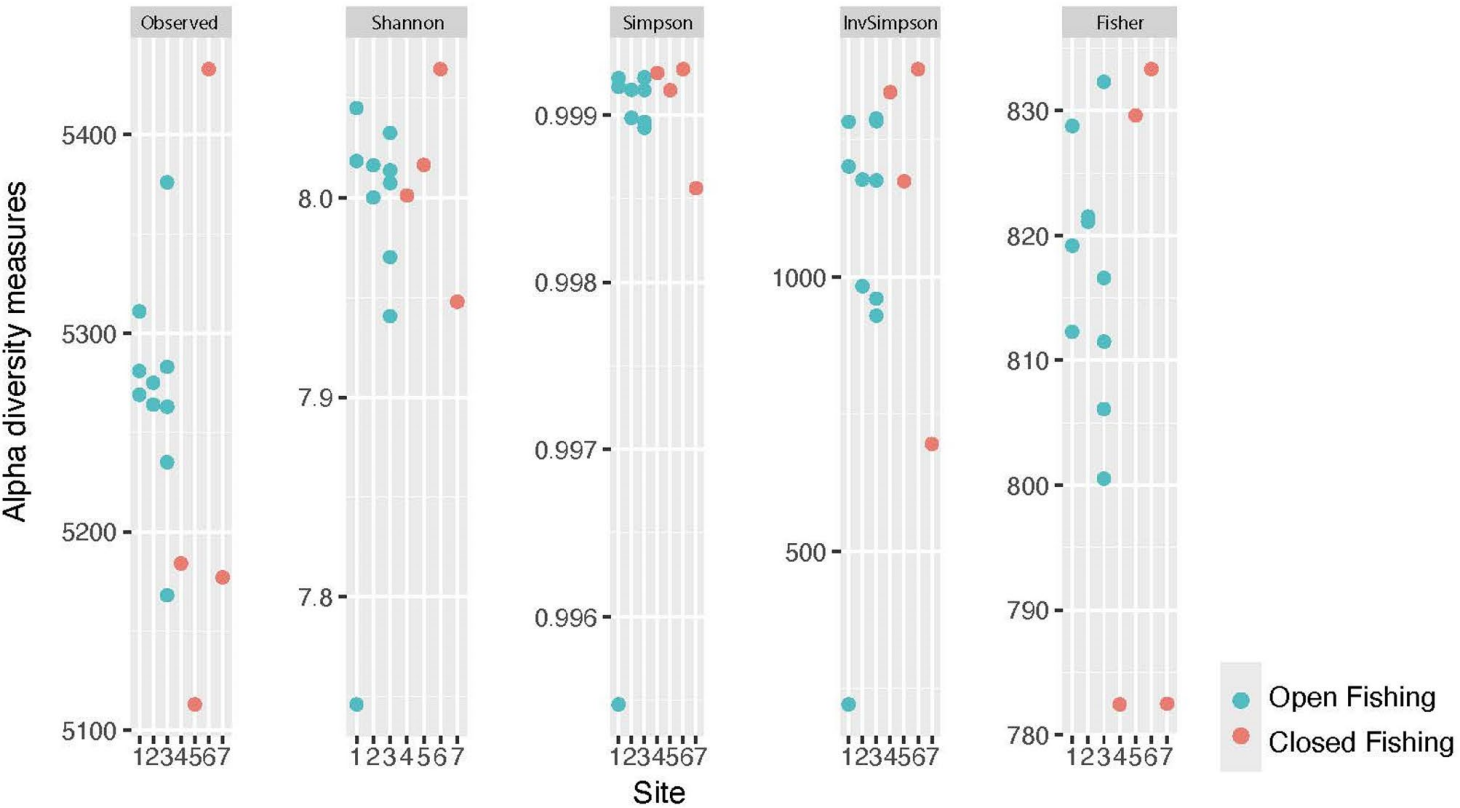
Alpha diversity measures based on species abundance across all sample sites organized by site.

Alpha diversity measures across sites and years varied minimally, ranging from 5113–5433 (observed), 7.75–8.06 (Shannon), 0.995–0.999 (Simpson), 221–1378 (InvSimpson), and 782–833 (Fisher) (Fig. 4). When viewing the measures plotted in the context of binary fishing exposure status (open vs. closed), no clear pattern emerged. Species richness ranged from 5178–5386 among sites open to fishing and from 5123–5441 among sites closed to fishing. Sites were classified according to the year and season collected, fishing exposure level and sediment type (Table 1). The NMDS Shepard plot displays the statistics for goodness of fit between ordination distances and observed dissimilarity (Fig. S1). A nonmetric fit R^2^ value of 0.988 and linear fit R^2^ value of 0.945 for fishing status were observed.

**Table 1:** Classifications by year and season (S; summer, F; fall) collected, site and the corresponding Shannon *H'* Index (Fig. 4a).

Site	Year	Area	Samples collected	Season collected	Fishing exposure level	Sediment type	Shannon index Summer	Shannon index fall
1	2017	Open	2	S + F	2	4	8.04	8.02
1	2019	Open	1	S	2	4	7.75	
2	2017	Open	2	S + F	2	4	8.0	8.0
3	2017	Open	2	S + F	1	4	8.0	8.0
3	2018	Open	1	S	1	4	7.94	
3	2019	Open	1	S	1	4	8.0	
3	2020	Open	1	S	1	4	7.97	
4	2018	Closed	1	S	0	3	7.99	
5	2018	Closed	1	S	0	3	8.02	
6	2019	Closed	1	S	0	2	8.06	
7	2019	Closed	1	S	0	2	7.95	

Fishing exposure level (0_minimal, 1_low, 2_moderate). Sediment type modified from the Barnhardt scale (1_Gravel, 2_Sand_gravel, 3_Sand_mud, 4_Mud_sand)^55^.

**Figure 5:**
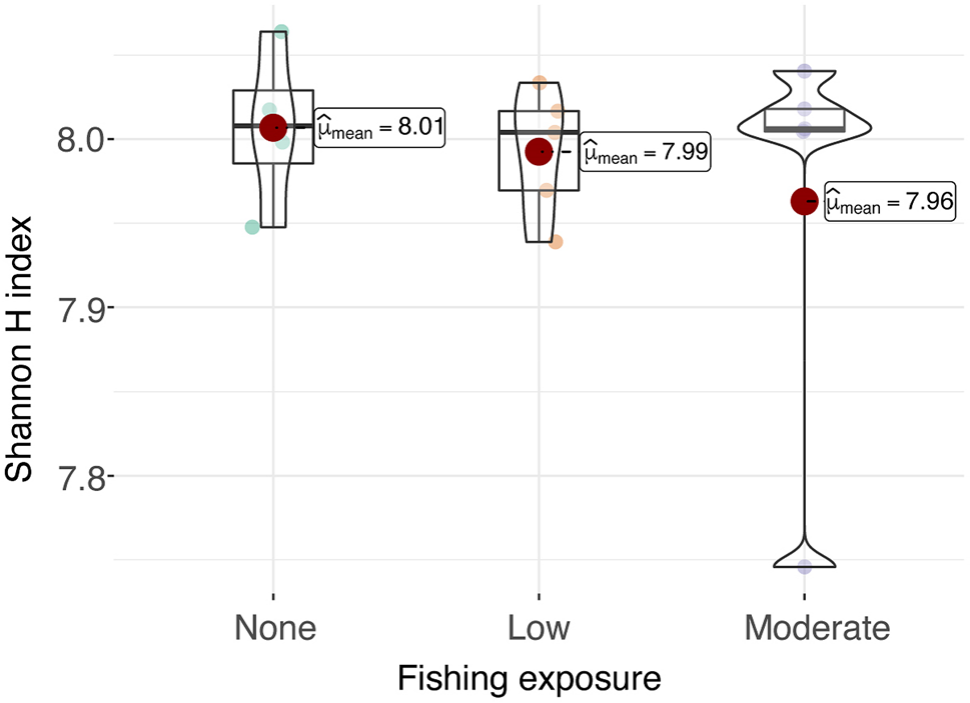
Boxplot of Shannon *H'*—Index by fishing exposure level (n = 14). The descriptive graphic of fishing exposure level is measured on an ordinal scale delineated as 0 (none), 1 (low), and 2 (moderate). Mean Shannon *H'* Index (from Table 1) is designated by the red circle.

The results from the multivariable models assessing associations between the Shannon *H'* Index and fishing exposure using the ordinal variable (none, low, moderate) suggested that higher levels of fishing exposure were marginally associated with lower richness after adjusting for temporal trends (n = 14; β = − 0.006 (SE 0.003); P = 0.0483) (Table 2). Time (year) was inversely associated with the Shannon *H'* index (β = − 0.006 (SE 0.002); P = 0.0207). Descriptive box plots of the ordinal fishing variable showed modest variation across fishing exposure levels (Fig. 5).

**Table 2:** Association between Shannon H-index and fishing exposure level.

Model parameter estimates^1^ (n = 14)					
Effect	Estimate (β)	Standard error	DF	t Value	P value
Intercept	14.120	4.460	11	3.17	0.0090
Fishing Exposure	− 0.006	0.003	11	− 2.22	0.0483
Year	− 0.006	0.002	11	− 2.70	0.0207

^1^SAS PROC GLIMMIX (version 9.4, Cary, N.C.) was employed to assess associations, using an inverse gaussian distribution and a log link function. Fishing exposure was measured on an ordinal scale delineated as 0 (none), 1 (low), 2 (moderate). *DF* degrees of freedom.

The results from the GLM assessing counts of species abundance in relation to the ordinal fishing exposure variable over time suggested that higher species abundance was associated with lower levels of fishing exposure (n = 83,804; β = − 0.057, SE 0.013, P < 0.0001 (95% confidence interval (CI) − 0.082, − 0.032); Table S2a. The model was adjusted for year, sediment type, and season. An inverse relationship was observed between year and abundance (β = − 0.075, SE 0.014, P < 0.0001 (95% CI − 0.102, − 0.048)). Generalized Linear Models are appropriate for skewed and clustered data structures because they specify the distribution of the observations, the linear predictor(s), a variance function, and a link function that can be specified by the user^53^. These features provide more efficiency and reduce bias in the variance parameters. In this study, models of species abundance (a count variable) utilized the zero-inflated negative binomial distribution with a logit link function (Table S2b)^54^.

### Binning of high-quality metagenomes and detection of BGCs

**Figure 6:**
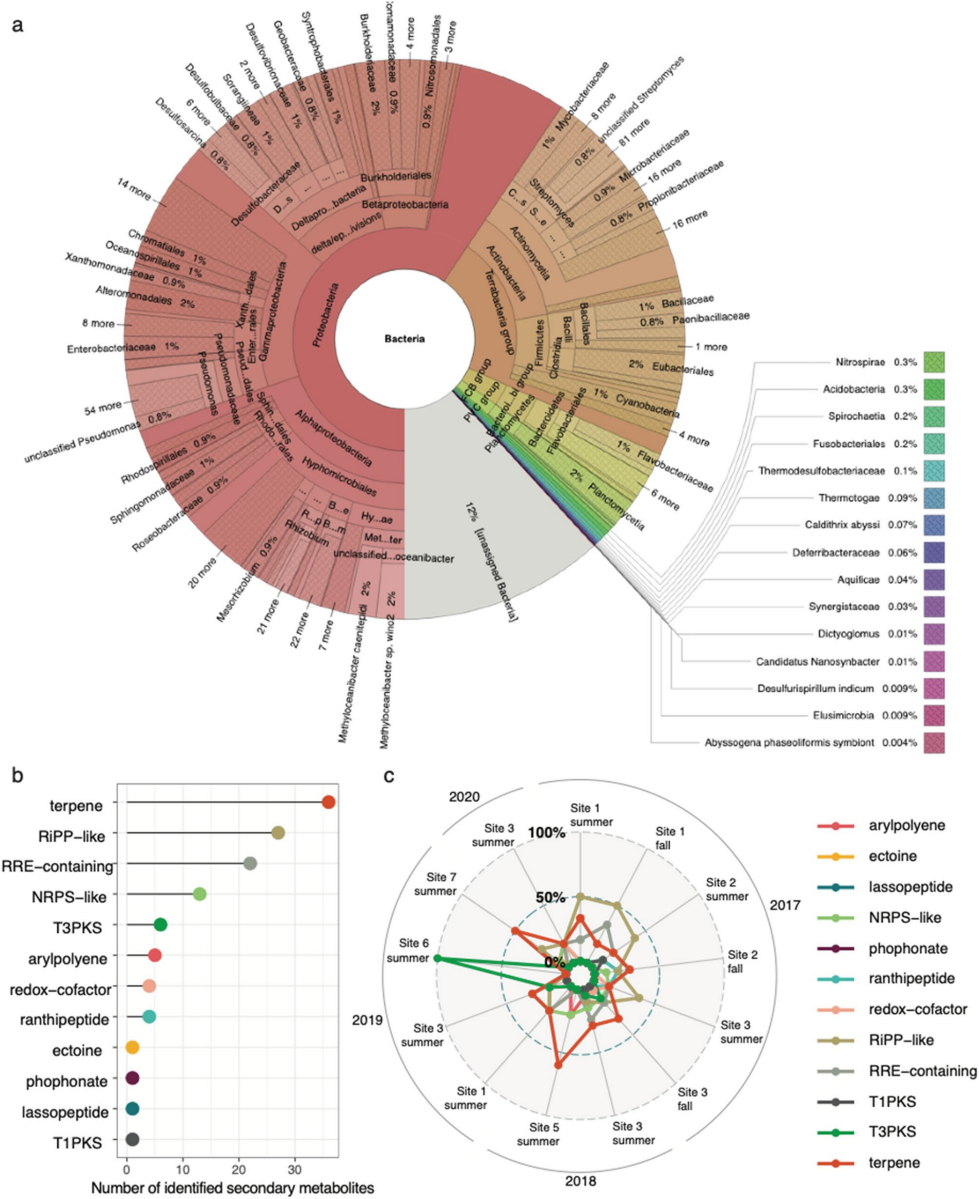
Taxonomic composition of high-quality metagenomes (MAGs) collected across all sample sites in this study, estimated with Kraken2 and visualized with KronaTools (**a**). Lollipop plots describing the number of BGCs detected across all sample sites (**b**). Radar plots exhibiting the proportion of secondary metabolites identified at each sample site (**c**).

Binning and refinement carried out with the metaWRAP binning module resulted in high-quality bins from 0 to 9 across the 14 samples, resulting in a total of 39,487 contigs. Site 4 collected in 2018 was the only sample that did not produce any bins with high-quality metagenomes (> 50% read complete, < 10% contamination). Combined analysis of the high-quality MAGs with Kraken2 resulted in the identification of 4402 different species across 1756 genera, 79 classes, and 41 phyla, with proportions largely reflecting those produced by abundance estimates (Fig. 6a). The results of the BGC analysis across all high-quality metagenomes resulted in the detection of 121 BGC sequences representing 12 different secondary metabolite clusters (Fig. 6b). Terpene was the most prevalent BGC, with 36 sequences identified, followed by RiPP-like BGCs (27 sequences) and RRE-containing BGCs (22 sequences). All other BGCs ranged from 1–13 sequences identified (overall mean = 10). The radar plot produced with ggRadar exhibits a wide range of proportions of each metabolite identified at each location, with no distinct trends (Fig. 6c). Nevertheless, RiPP-like secondary metabolites appear to make up a comparatively large proportion of BGCs identified at site 1 in 2017, while terpene exhibits the same pattern at site 5 in 2018 and site 7 in 2019.

### Environmental indicators

Sediment samples were classified into four main categories based on the Barnhardt scale^55^, which consisted of  ≥ 90% of a particular sediment type. Descriptive indicators of water quality trends were evaluated in relation to metagenomic results by site, collection year, fishing exposure, season, and sediment type. Proximal to the open sites, seasonal trends in water temperature and salinity were observed, as both parameters were higher in the fall than in the summer (Table S3). More research is needed to determine whether this result reflects environmental stress to the seafloor habitat, particularly if the levels are above threshold limits for extended periods of time^56^.

## Discussion

The spatial distribution of microbial diversity in marine sediment within a continental shelf region is not well documented.^1^ For SBNMS managers, a primary focus of habitat quality is the assessment of benthic habitats. The present study is unique as the first time-series report that provides a metagenomic assessment of abundance, biodiversity, and fishing exposure for sediment microbial communities in a National Marine Sanctuary. As a working sanctuary, the SBNMS is a critical fisheries resource, providing ecosystem services for conservation management^57^. The results underscore the need to improve our understanding of microbial habitat quality in marine protected areas. For example, the trends observed in time and fishing exposure levels highlight the fact that MPAs should be monitored more frequently to assess potentially significant changes in structure and function to support a robust food web^58^. Environmental stressors such as fishing activity may reduce biodiversity over time^59^; however, more study is needed to elucidate these complex trends within the benthic invertebrate habitat.

In terms of taxonomic abundance trends, classes Gamma-, Alpha-, Beta-, and Deltaprotobacteria were the dominant taxa, followed by Actinomycetia across all sites. Similar to previous SBNMS reports evaluating coastal sediment communities,^7,9^ these taxa serve to maintain habitat quality by cycling carbon and processing nutrients^60^. In terms of genera, *Pseudomonas* and *Streptomyces* were the two most dominant types observed. Actinobacteria was also prevalent and has great potential to produce abundant BGCs associated with antimicrobial agents^61^. The results are consistent with previous research within the SBNMS, which identified class Actinobacteria and species *Streptomyce*s within the open fishing area^62^. Within the SBNMS, there were 4 sites that showed 50% or greater key secondary metabolites, such as terpene, RiPP-like and T3PKS. These sites were sites 1 and 5, 6 and 7. *Streptomyces* species were relatively abundant, with 236 different strains identified. The combined use of MAGS with antiSMASH exhibited high per-sequence accuracy and enabled BGC screening that showed a wide distribution of potential medically relevant secondary metabolites. The BGC analysis was performed on binned genomes that were nearly complete. Future research to detect diversity and further BGC characterization within strains is needed, as improved computational methods can inform metagenomic studies^63^.

The time-series assessment of the taxonomic structure illustrated in the present report fill a gap in the literature for the GoM. Compared to metagenomic studies of oceanic sediments, the biodiversity results from this SBNMS study are relatively similar to continental shelf sediment studies in temperate and subtropical zones at similar depths^64^. However, relative abundance and diversity measures should be taken in context with respect to surficial versus subseafloor habitat and bioinformatics methods.

Environmental factors such as water temperature suggested a potential restructuring of the microbial community from summer to fall as bottom temperatures increased. Although the seasonal conditions comply with an observable trend for this region^65^, it is difficult to assess the status of benthic communities due to the lack of consistent water quality monitoring data. Potential water quality impacts on benthic habitats may be associated with variable occurrences of vessel traffic, which can impact microbial communities by trawling activities that resuspend soft sediments and increase turbidity in bottom water habitats^66^. Although warmer water from the Gulf Stream current regularly influences the GoM during the fall, observations of increased warming and higher salinity levels may contribute to shifts in microbial communities.^66^ Further study is recommended to assess potential shifts in bacterial assemblages since indirect effects of water temperature^67,68^ and ocean currents may be influential.

In the present study, species diversity as measured by the Shannon *H'* index was marginally higher among sites that were closed to fishing than among sites with minimal or moderate levels of fishing activity. Multivariable models assessing counts of species abundance in relation to the ordinal fishing expsoure variable over time suggested that higher species abundance was associated with lower levels of fishing exposure after adjusting for year and sediment type. However, more detailed assessments of fishing activity using continuous variables from VMS data should also be conducted as more data become available. These results underscore the need to monitor the microbial community structure and diversity across the full range of fishing activities, including areas where heavier fishing vessel traffic is more consistent.

The present assessment was limited by the relatively small number of sampling units analyzed and the fact that areas with heavy fishing activity were not sampled. Resource capacity limitations precluded sampling in heavier fishing activity zones that may be exposed to more disruptive bottom gear. Furthermore, the intensity of fishing activity may vary by season and location. Sampling additional sites would have afforded a more powerful assessment of biodiversity trends by site, season, and fishing activity. Another limitation is that the VMS data do not capture data from recreational fishing vessels. Additionally, water quality data from in situ sensors at depth would provide information to assess potential abundance and biodiversity shifts with respect to fishing impact.

Prior research conducted in the northwest corner of Stellwagen Bank documented periodic impacts from fishing exposure^6,7^. However, it is unclear whether reductions in fishing effort have resulted in reduced risk to healthy populations of higher trophic level organisms (i.e., seabirds, cod, and right whales). Multiple stressors such as pollution and climate change continue to affect these habitats^69^. In the present study, the data trended toward higher species abundance and higher average biodiversity values in areas with lower levels of fishing activity. This is consistent with NOAA reports documenting lower levels of direct impact in the Western Gulf of Maine Closure Area, suggesting that closure has supported recovery in some areas^70^. Further analyses with a wider range of sites proximal to monitoring stations and inclusive of areas exposed to heavier fishing activity are needed to assess whether statistically significant trends in microbial community abundance and biodiversity can be consistently observed.

In conclusion, closure areas are critical to evaluating the biodiversity of the microbial benthic habitat, since long-term monitoring is not routinely conducted. The present study revealed important public health implications with respect to protecting fragile marine ecosystems. As recognized by the United Nations Sustainable Development Goals (SDG #17, Life Below Water), marine sanctuaries represent a vast oceanic resource and provide myriad ecosystem services that are important for human health^71^. These services include the potential for harboring secondary metabolites for natural products and drug discovery^72^. Collectively, this research underscores the importance of raising awareness about the life-supporting functions of marine sanctuaries and the possibility of engaging citizen scientists and health professionals in the stewardship of these resources.

## Supplementary Information

Supplementary Information.

## Data Availability

All the metagenomic raw sequencing data from this study has been deposited in the NCBI Sequence Read Archive (SRA). The BioProject accession number for the 2017 data are PRJNA524407 (BioSample SAMN10999726-SAMN10999731) and PRJNA837619 (BioSample SAMN28209579-28209594) for 2018–2020.
