# Supplementary material for: Metagenomics to characterize sediment microbial biodiversity associated with fishing exposure within the Stellwagen Bank National Marine Sanctuary
Source: Sci Rep. 2022 Jun 9;12:9499. doi: 10.1038/s41598-022-13409-5 (PMC9184631; doi:10.1038/s41598-022-13409-5)
Supplement: Supplementary file 1 — Supplementary Information. [file 41598_2022_13409_MOESM1_ESM.pdf]

Supplementary Material

Metagenomics to characterize sediment microbial biodiversity associated with fishing exposure within the Stellwagen Bank National Marine Sanctuary

Authors

Spencer A. Bruce[1], Semra A. Aytur[2], Cheryl P. Andam[1], John P. Bucci [3*, 4]

Affiliations

1. Department of Biological Sciences, University at Albany, State University of New York, Albany, NY, 12222, USA.
2. Department of Health Management & Policy, University of New Hampshire, Durham, NH, 03824, USA.
3*. School of Marine Science & Ocean Engineering, University of New Hampshire, Durham, NH, 03824, USA.
4. Marine Microverse Institute, Kittery Point, ME, 03905, USA.

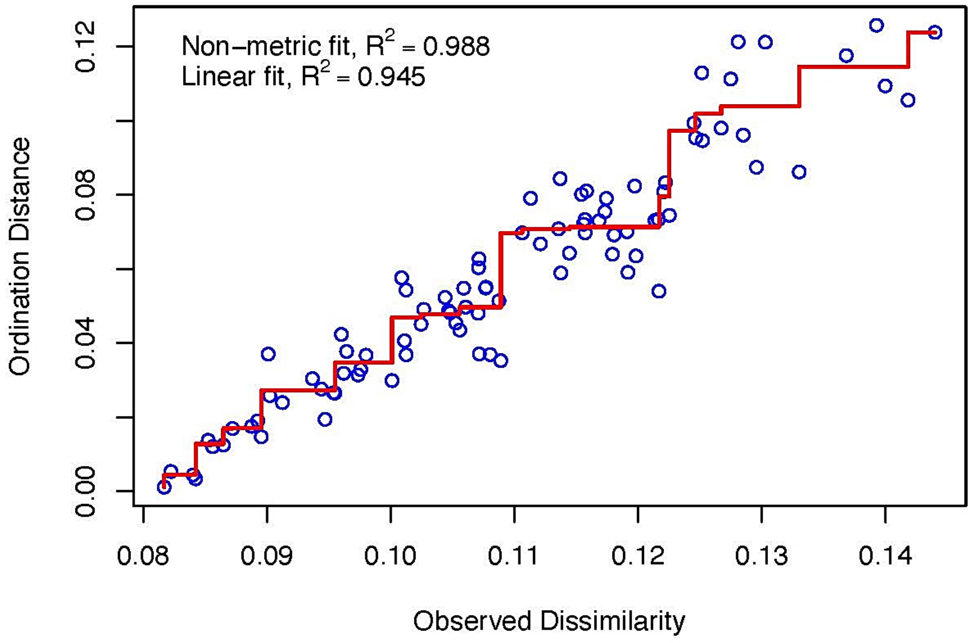

Figure S1. NMDS Shepard plot displaying the statistics for goodness of fit between ordination distances and observed dissimilarity. A nonmetric fit R^2 value of 0.988 and linear fit R^2 value of 0.945 for fishing status were observed.

Table S1. From the read data, Bracken estimation of abundance at multiple taxonomic levels by site showed sites 7 and 4 to have highest mean value.

Site	Mean value	Standard Deviation
1	86.49	277.92
2	83.77	83.77
3	87.90	184.10
4	98.44	183.62
5	65.68	132.97
6	94.30	172.42
7	97.51	268.97

Table S2a. Generalized linear model[1] of the association between species abundance (count) (n=83,804) and fishing exposure level, adjusted for covariates. Fishing exposure was measured on an ordinal scale delineated as 0 (none), 1 (low), 2 (moderate). Zero-inflated negative binomial models are appropriate for modeling count variables with excessive zero values. The two parts of the zero-inflated model are a binary model to assess the likelihood of a zero outcome, and a count model (negative binomial) to model the count process.

Parameter Estimates						
**Parameter**	**Estimate (β)**	**Standard Error**	**Wald 95% Confidence Limits**		**Wald Chi-Square**	**P Value**
**Intercept**	23.428	8.442	6.882	39.974	7.70	0.0055
**Year**	-0.009	0.004	-0.017	-0.001	4.97	0.0258
**Fishing Exposure**	-0.019	0.003	-0.025	0.013	36.19	<0.0001
**Season**	0.013	0.010	-0.007	0.032	1.66	0.1978
**Zero Inflation Parameter Estimates**						
**Parameter**	**Estimate (β)**	**Standard Error**	**Wald 95% Confidence Limits**		**Wald Chi-Square**	**P Value**
**Intercept**	148.942	28.028	94.007	203.876	28.24	<0.0001
**Fishing Exposure**	-0.057	0.013	-0.082	-0.032	19.79	<0.0001
**Year**	-0.075	0.014	-0.102	-0.048	29.04	<0.0001
**Sediment Type**	0.026	0.021	-0.015	0.066	1.53	0.2161
**Season**	0.064	0.032	0.002	0.126	4.07	0.0438

[1]Generalized linear models were run using SAS PROC GENMOD (version 9.4, Cary, N.C.) with a zero-inflated negative binomial distribution and a logit link function.

Table S2b. Sensitivity Analysis: Generalized linear model [1] of the association between species abundance (count) (n=65,846) and fishing exposure level, restricted to the summer season. The main effects for fishing exposure and time remain statistically significant. Models were robust to specification.

Parameter Estimates						
**Parameter**	**Estimate (β)**	**Standard Error**	**Wald 95% Confidence Limits**		**Wald Chi-Square**	**P Value**
**Intercept**	22.273	8.607	5.403	39.142	6.70	0.0097
**Year**	-0.009	0.004	-0.017	-0.0004	4.21	0.0402
**Fishing Exposure**	-0.018	0.004	-0.025	-0.0109	26.04	<0.0001

Zero Inflation Parameter Estimates						
**Parameter**	**Estimate (β)**	**Standard Error**	**Wald 95% Confidence Limits**		**Wald Chi-Square**	**P Value**
**Intercept**	156.617	28.294	101.1609	212.0729	30.64	<0.0001
**Fishing Exposure**	-0.074	0.015	-0.1030	-0.0445	24.50	<0.0001
**Year**	-0.079	0.014	-0.1061	-0.0512	31.48	<0.0001
**Sediment Type**	0.042	0.022	-0.0011	0.0853	3.64	0.0563

Table S3. Water quality parameters showing the mean of daily averages by season each year collected at the NERACOOS Massachusetts Bay Buoy (A01-50 m) station proximal to open and Jeffrey's Ledge (JL) (#44098 - surface) proximal to closed fishing areas. National Data Buoy Data Center daily averages were obtained from sensors. The A01 station is within 10 nautical miles of the open region (sites 1-3). The JL buoy is located approximately 20 nautical miles (37 km) from the closed region (sites 4-7) and measures surface water only.

	Summer Water Temp F	Fall Water Temp F	Annual Water Temp F	
2017	42.11 ± 1.20	48.22 ± 5.72	41.88 ± 3.30	
2018	46.33 ± 2.50	49.59 ± 0.76	44.26 ± 4.20	
2019	42.93 ± 0.32	47.33 ± 0.87	41.58 ± 2.79	
2020	44.57 ± 0.96	47.49 ± 1.25	44.33 ± 2.28	
	Dissolved Oxygen ml/l	Dissolved Oxygen ml/l	Dissolved Oxygen ml/l	
2017	5.07 ± 0.36	6.20 ± 0.34	5.58 ± 0.31	
2018	5.69 ± 0.28	5.60 ± 0.37	5.62 ± 1.31	
2019	6.17 ± 0.12	3.91 ± 0.32	5.24 ± 1.26	
2020	3.15 ± 0.11	5.04 ± 0.95	4.24 ± 0.98	
	Salinity psu	Salinity psu	Salinity psu	
2017	31.78 ± 0.13	32.37± 0.12	32.33 ± 0.39	
2018	32.13 ± 0.13	32.56 ± 0.16	32.40 ± 0.30	
2019	31.83 ± 0.04	32.60 ± 0.18	32.21 ± 0.36	
2020	32.04 ± 0.11	32.29 ± 0.19	32.24 ± 0.24	
	Chlorophyll a ug/l	Chlorophyll a ug/l	Chlorophyll a ug/l	
2017	2.81 ± 1.87	4.97 ± 1.65	6.50 ± 5.05	
2018	2.98 ± 1.48	2.82 ± 1.10	4.43 ± 3.78	
2019	3.46 ± 1.27	1.42 ± 0.51	2.10 ± 1.85	
2020	n/a	4.32 ± 1.62	3.20 ± 2.06	
	Summer A01 1m Water Temp F	Annual A01 1m Water Temp F	Summer Jeffrey's Ledge 1m Water Temp F	Annual Jeffrey's Ledge 1m Water Temp F
2017	59.49 ± 1.9	45.87 ± 5.92	63.84 ± 3.3	53.93 ± 8.82
2018	n/a	n/a	67.44± 3.01	53.18 ± 10.54
2019	n/a	n/a	n/a	n/a
2020	n/a	n/a	67.0 ± 2.71	54.65 ± 10.18

n/a = sensor data unavailable.
